# COmbinatioN effect of FInerenone anD EmpaglifloziN in participants with chronic kidney disease and type 2 diabetes using a UACR Endpoint (CONFIDENCE) trial: baseline clinical characteristics

**DOI:** 10.1093/ndt/gfaf022

**Published:** 2025-02-07

**Authors:** Rajiv Agarwal, Jennifer B Green, Hiddo J L Heerspink, Johannes F E Mann, Janet B McGill, Amy K Mottl, Julio Rosenstock, Peter Rossing, Muthiah Vaduganathan, Meike Brinker, Robert Edfors, Na Li, Markus F Scheerer, Charlie Scott, Masaomi Nangaku, for the CONFIDENCE investigators, Agostino Consoli, Ahmed Awad, Alberto Ortiz Arduan, Alfonso Soto, Ali Iranmanesh, Amy K Mottl, An Nollet, Ankur Doshi, Anna Maria Grazia Veronelli, Architkumar Patel, Ashar Luqman, Balasubramaniyan T, Bernhard Winkelmann, Bruce Baker, Bruno Guerci, Bruno Van Vlem, Bruno Verges, Byung Wan Lee, Carolina Solis-Herrera, Chandrashekar Matad, Chang Beom Lee, Chien-Te Lee, Chiz-Tzung Chang, Choon-Hee Chung, Christof Kloos, Christoph Axthelm, Claus Juhl, Cristina Castro, Cristobal Morales, Csaba Kovesdy, Daishiro Yamada, Dana Mitchell, David Gaskin, David LaMond, Der-Cherng Tarng, Dinesh Khullar, Pierre-Louis Carron, Manisha Sahay, Elie Sahyouni, Emanuele Bosi, Enrico Fiaccadori, EunYoung Lee, Faiad Adawi, Fernando Cereto Castro, Francis Duyck, Francisco Martinez Deben, Francisco Tinahones Madueno, Fumi Umeoka, Ganapathi Bantwal, Genya Aharon-Hananel, German Hernandez, Giancarlo Tonolo, Giuseppe Mazza, Giuseppe Penno, Gloria Ortiz, Guillermo Umpierrez, Hanane Bourarich, Hansraj Alva, Harold Miller, Harvey Serota, Hideo Kanehara, Hidetoshi Kanai, Hitesh Mehta, Idit Liberty, Iqbal Khalid, Jae-Myung Yu, Jared Probst, Jay Sandberg, Jay Shubrook, Jayakumar EK, Jean-Pierre Fauvel, Jeroen van der Net, Jesper Nørgaard Bech, Jose Luis Górriz Teruel, Jose Mandry, Joseph Ravid, Juan Diego Mediavilla, Jugal Bihari Gupta, Julie Silverstein, Julio Wainstein, Ju-Ying Jiang, Keshavamurthy CB, Keung Lee, Klaus Busch, Kunihisa Kobayashi, Leslie Spry, Lutz Stemler, Mai-Szu Wu, Maria Jose Soler Romeo, Maria Marques Vidas, Mariana Garcia-Touza, Marijn Speeckaert, Markus van der Giet, Masahiko Ochi, Masao Ishii, Matthew Ray, Mazen Elias, Minesh Rajpal, Ming Ju Wu, Mirjam Lips, Mohamed El-Shahawy, Nauman Shahid, Nimer Assy, Nomy Levin-Iaina, Olivier Dupuy, Olivier Moranne, Osvaldo Brusco, Pablo Pergola, Pal Atanu, Paola Ponzani, Paul Rootjes, Pedro Velasquez Mieyer, Peter Doubel, Peter Luik, Peter Rossing, Pieter Gillard, Piotr Lazowski, Prabha Dadala Ratna, Raj Singh, Rekha John, Richard Powell, Richard Tytus, Roberta Poli, Roberto Cimino, Roberto Trevisan, Salvatore De Cosmo, Sameer Chaubey, Sameh Fikry, Sanjay Chunilal Agarwal, Saurabh Agarwal, Scott Hines, Sean Peterson, Seok Joon Shin, Sharma Balram, Shih-Te Tu, Shivinder Jolly, Siddharth Mavani, Soo Lim, Sree Bhushan Raju, Sreedhar Reddy, Steve Fordan, Subhash Wangnoo, Sung-Gyun Kim, Syed Pervaiz, Takeshi Osonoi, Terumasa Hayashi, Thorsten Koch, Thure Krarup, Tuan-Huy Tran, Tushar Bandgar, Vernekar Ritesh, Veronica Resi, Wajdi Al-Shweiat, Wayne Kotzker, William Beaubien-Souligny, William Kaye, William Yang, Woo-Je Lee, Yoshihide Hirohata, Yoshimitsu Yamasaki, You-Cheol Hwang, Young Min Cho, Young Sun Kang

**Affiliations:** Division of Nephrology, Department of Medicine, Richard L. Roudebush VA Medical Center & Indiana University School of Medicine, Indianapolis, IN, USA; Division of Endocrinology, Department of Medicine and Duke Clinical Research Institute, Duke University School of Medicine, Durham, NC, USA; Department of Clinical Pharmacy and Pharmacology, University Medical Center Groningen, Groningen, The Netherlands; KfH Kidney Centre, Munich, Germany; Department of Nephrology & Hypertension, Friedrich Alexander University, Erlangen, Germany; Division of Endocrinology, Metabolism & Lipid Research, Washington University School of Medicine in St. Louis, St Louis, MO, USA; University of North Carolina Kidney Center, UNC School of Medicine, Chapel Hill, NC, USA; Velocity Clinical Research at Medical City, Dallas, TX, USA; University of Texas Southwestern Medical Center, TX, USA; Steno Diabetes Center Copenhagen, Copenhagen, Denmark; Department of Clinical Medicine, University of Copenhagen, Copenhagen, Denmark; Division of Cardiovascular Medicine, Brigham and Women's Hospital and Harvard Medical School, Boston, MA, USA; Cardiology and Nephrology Clinical Development, Bayer AG, Wuppertal, Germany; Department of Clinical Sciences, Danderyd University Hospital, Division of Cardiovascular Medicine, Karolinska Institutet, Stockholm, Sweden; Bayer Healthcare, Chaoyang District, Beijing, China; Global Medical Affairs and Pharmacovigilance, Bayer AG, Berlin, Germany; Clinical Statistics and Analytics, Bayer PLC, Reading, UK; Division of Nephrology and Endocrinology, The University of Tokyo Graduate School of Medicine, Tokyo, Japan

**Keywords:** chronic kidney disease, clinical trial, finerenone, sodium–glucose cotransporter 2 inhibitor, type 2 diabetes

## Abstract

**Background:**

Finerenone, a selective nonsteroidal mineralocorticoid receptor antagonist, and sodium–glucose cotransporter 2 inhibitors (SGLT2is) both reduce chronic kidney disease (CKD) progression and improve kidney/cardiovascular (CV) outcomes. The CONFIDENCE (COmbinatioN effect of FInerenone anD EmpaglifloziN in participants with chronic kidney disease and type 2 diabetes using a UACR Endpoint) study (NCT05254002; EudraCT 2021-003037-11) hypothesis is that early combination of finerenone and empagliflozin, an SGLT2i, is superior to either drug alone in reducing urine albumin-to-creatinine ratio (UACR) over 6 months.

**Methods:**

CONFIDENCE is an ongoing, fully enrolled, randomized, controlled, double-blind, multicentre phase 2 clinical trial in adults (≥18 years of age) with CKD and type 2 diabetes (T2D), estimated glomerular filtration rate (eGFR) of 30–90 mL/min/1.73 m^2^ and UACR of ≥100 to <5000 mg/g. Participants taking the clinically maximum tolerated dose of a renin–angiotensin system inhibitor for >1 month at screening were eligible. Participants were randomized 1:1:1 to once-daily finerenone plus empagliflozin, finerenone plus placebo, or empagliflozin plus placebo; doses were 10 mg once daily for empagliflozin and 10 or 20 mg once daily for finerenone, depending on eGFR at baseline. Randomization was stratified by eGFR (<60 or ≥60 mL/min/1.73 m^2^) and UACR (≤850 or >850 mg/g). The primary efficacy outcome is the relative change in UACR from baseline at Day 180.

**Results:**

There were 818 participants randomized across 143 sites from 14 countries between July 2022 and August 2024. Mean (standard deviation) eGFR was 54.2 (17.1) mL/min/1.73 m^2^. Median (interquartile range) UACR was 583 (292, 1140) mg/g. Mean (standard deviation) HbA1c was 7.3 (1.2)%. Mean systolic/diastolic blood pressure was 135.2/77.3 mmHg. Glucagon-like peptide-1 receptor agonists and insulin were used by 182 (23%) and 313 (39%) participants, respectively. Atherosclerotic CV disease, diabetic retinopathy and a history of heart failure were present in 223 (28%), 126 (16%) and 30 (4%) participants, respectively.

**Conclusions:**

The CONFIDENCE trial enrolled a diverse population with CKD and T2D, and will determine the impact of simultaneous initiation of combination finerenone and an SGLT2i versus individual therapy on potentially mitigating the progression of CKD in people with T2D.

**Trial registration number:**

ClinicalTrials.gov NCT05254002; EudraCT 2021-003037-11.

## Graphical Abstract

**Graphical Abstract:**
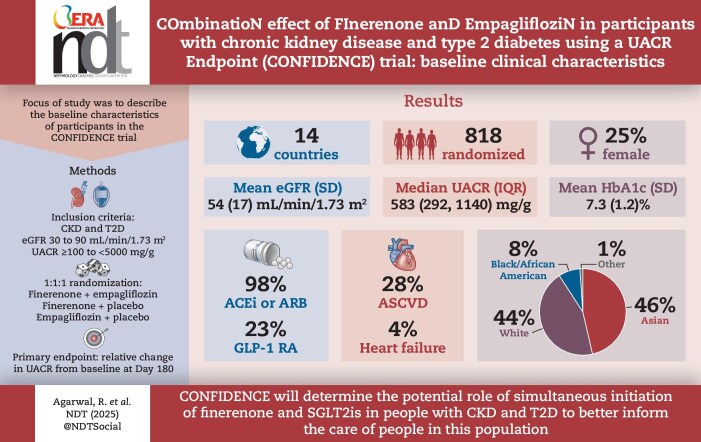

KEY LEARNING POINTS **What was known**:

- Chronic kidney disease (CKD) in type 2 diabetes (T2D) increases the risk of kidney failure and, compared with T2D alone, may more than double the rate of cardiovascular (CV) morbidity and all-cause mortality risk.
- Finerenone and sodium–glucose cotransporter 2 inhibitors (SGLT2is) reduce adverse kidney and CV outcomes in people with CKD and T2D.

**This study adds**:

- CONFIDENCE enrolled a contemporary cohort of participants with CKD and T2D at high risk of kidney disease progression and CV events.
- CONFIDENCE is the first randomized controlled trial to examine the effect of initial simultaneous treatment with a nonsteroidal mineralocorticoid receptor antagonist and an SGLT2i, versus either agent alone, on urine albumin-to-creatinine ratio and safety outcomes.

**Potential impact**:

- The CONFIDENCE trial will determine the safety of simultaneous initiation, as well as the benefit of dual therapy, of finerenone and SGLT2is in people with CKD and T2D to better inform the care of people in this population.

## INTRODUCTION

Chronic kidney disease (CKD) affects approximately 9% of the population worldwide [1], with diabetes being a leading cause of CKD [2–4]. CKD is associated with an increased risk for premature death, dialysis and major cardiovascular (CV) outcomes, accounting for 1.2 million deaths globally in 2017 [1, 5]. Individuals with a combination of kidney disease and type 2 diabetes (T2D) have greater mortality rates compared with those with T2D alone [6]. The risk of mortality increases linearly when an individual's estimated glomerular filtration rate (eGFR) is below a threshold of approximately 60 mL/min/1.73 m^2^ and albuminuria levels are increased [7].

The 2024 Kidney Disease: Improving Global Outcomes (KDIGO) guideline and the American Diabetes Association recommend several drugs to reduce the risks of CKD, including renin–angiotensin system inhibitors, sodium–glucose cotransporter 2 inhibitors (SGLT2is), the selective nonsteroidal mineralocorticoid receptor antagonist finerenone, and a glucagon-like peptide-1 receptor agonist (GLP-1 RA) with demonstrated benefit in a population with CKD and T2D [8, 9]. Of note, the efficacy and safety of the approved therapies for the management of CKD have been studied in individual placebo-controlled trials. However, the combined effects on important CKD outcomes, such as albuminuria, are mainly untested.

While contemporary trials in people with CKD have required the use of maximally tolerated angiotensin-converting enzyme inhibitors (ACEis) or angiotensin receptor blockers (ARBs) as background therapy, detailed data on the initiation and combination of therapies are lacking for individuals with CKD and T2D, especially on whether these drugs should be sequentially or simultaneously initiated. Secondary analyses of FIDELITY [a pooled analysis of the FIGARO-DKD (NCT02540993) and FIDELIO-DKD (NCT02545049) trials] suggested a potential additive effect of finerenone and SGLT2is on kidney and CV outcomes in a subset of participants with CKD and T2D [10]. The potential for an additive effect of finerenone and SGLT2is on kidney and CV outcomes in T2D is supported by their distinct, yet complementary, mechanisms of action [11]. Using population pharmacokinetics and pharmacodynamics in a pooled analysis of FIGARO-DKD and FIDELIO-DKD (FIDELITY), Eissing *et al*. provided evidence for a potential additive effect of finerenone with an SGLT2i on urine albumin-to-creatinine ratio (UACR)-lowering and chronic eGFR slope [12]. However, no trial has yet evaluated the safety and efficacy of simultaneously initiating an SGLT2i and finerenone in people with T2D and albuminuria.

The COmbinatioN effect of FInerenone anD EmpaglifloziN in participants with chronic kidney disease and type 2 diabetes using a UACR Endpoint (CONFIDENCE; NCT05254002; EudraCT 2021-003037-11) trial is investigating whether early combination therapy comprising finerenone and the SGLT2i empagliflozin is superior to either drug alone in reducing UACR [11, 13, 14]. To our knowledge, CONFIDENCE is the first randomized controlled trial examining the safety, including on initial combined haemodynamic effects, and efficacy of combination therapy comprising finerenone and an SGLT2i for the treatment of CKD and T2D. Here we report the baseline characteristics of the randomized trial participants.

## MATERIALS AND METHODS

### Study design

The study rationale and design of CONFIDENCE have previously been reported [11]. Briefly, CONFIDENCE is an ongoing, fully enrolled, randomized, controlled, double-blind, double-dummy, global, multicentre, phase 2 clinical trial in people (≥18 years of age) with CKD and T2D. There has been one key amendment made to the CONFIDENCE trial protocol, dated 24 October 2023, to increase generalizability and enrolment. The most clinically relevant change was related to study eligibility; specifically, the lower limit of the UACR inclusion criterion was amended to allow enrolment of participants with a lower range of UACRs (≥100 mg/g instead of previously ≥300 mg/g).

As finerenone and empagliflozin both moderately decrease blood pressure and eGFR after initiation of treatment [15–18], their combined effect on blood pressure (using 24-h blood pressure monitoring) and other safety parameters were assessed by the Internal Displacement Monitoring Centre in a subgroup of participants with eGFR between 40 and 90 mL/min/1.73 m^2^, prior to extending enrolment to include participants with eGFR as low as 30 mL/min/1.73 m^2^ [11]. As such, CONFIDENCE comprised two consecutive parts. In Part A, participants with an eGFR of 40–90 mL/min/1.73 m^2^ were enrolled and underwent ambulatory blood pressure monitoring (ABPM) for 24 h after administration of their first study drug dose on Day 1. To monitor the risk of hypotension, all participants included in Part A were equipped with an ABPM device 1 h before the first intake at the study sites, remained at the study site for 4–6 h for office blood pressure monitoring, and kept the ABPM device on for 24 h. eGFR was monitored at each study visit, with a first assessment 14 days after the initial intake. A decision to progress from Part A to Part B, which includes extension of enrolment to participants with an eGFR as low as 30 mL/min/1.73 m^2^, was made by the sponsor and steering committee following a blinded analysis of safety findings for the participants in Part A and an unblinded review by the independent data monitoring committee. In Part B, participants were not required to undergo ABPM.

**Figure 1:**
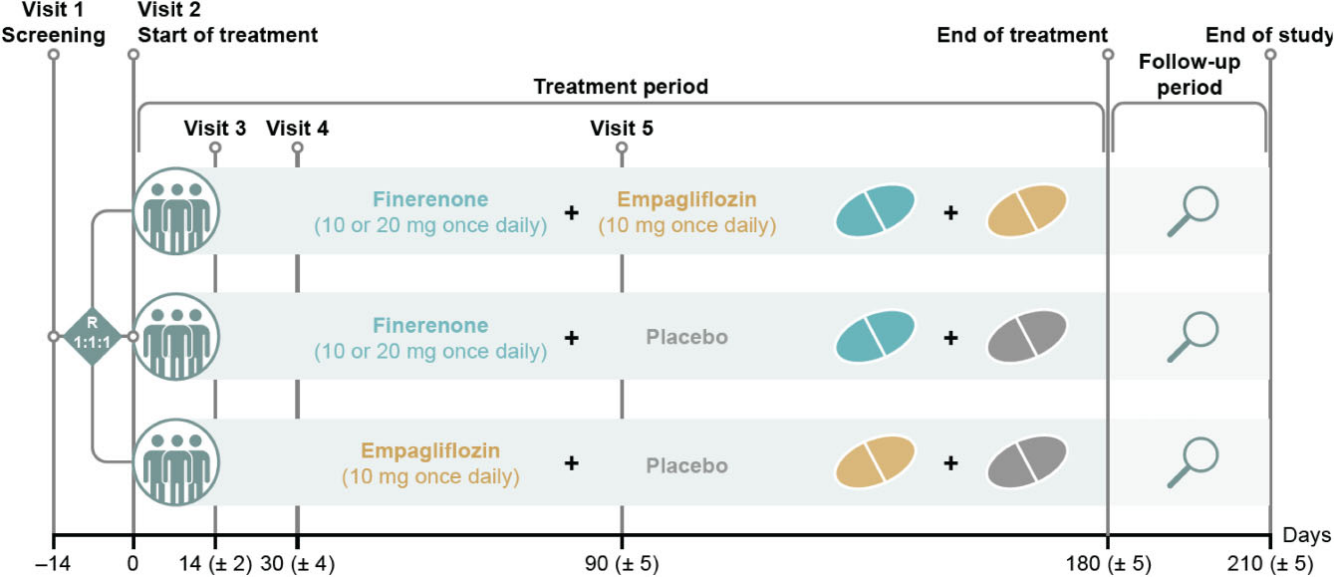
Study design. The number of participants will be capped in Parts A and B as follows: <80% with eGFR ≤75 mL/min/1.73 m^2^ and <20% with eGFR >75 mL/min/1.73 m^2^. Up/downtitration based on eGFR, serum/plasma potassium or potassium, safety and tolerability. R, randomization. Adapted from Green *et al.* [11] under the terms of the CC-BY-NC 4.0 license.

Participants had to have T2D with a glycated haemoglobin (HbA1c) level of <11% at screening and be treated with the maximum tolerated dose of an ACEi or ARB for >1 month at their screening visit (Visit 1). Participants also had to have a UACR of ≥100 to <5000 mg/g and an eGFR of 30–90 mL/min/1.73 m^2^ (Fig. 1).

Demographic characteristics, medical history, and prior and concomitant medication were documented for participants at the screening visit. Prior and concomitant medications were also documented at Day 1 before the allocation of the study drug. Demographic characteristics included age, sex, race, ethnicity, geographic region, body height and smoking history. Other measurements at screening included UACR, creatinine-based eGFR [the 2009 Chronic Kidney Disease Epidemiology Collaboration (CKD-EPI) equation was used for eGFR [19] with a modification (year 2010) to the equation for Japanese participants [20] and serum potassium. Historical values for HbA1c were acceptable providing they were obtained within 3 months of the screening visit. Medical history was coded using MedDRA. Screening visits were used for assessing study eligibility only. Participants with certain medical conditions were excluded, including those with bilateral clinically relevant kidney artery stenosis (>75%) or other nondiabetic kidney disease, heart failure with reduced ejection fraction (New York Heart Association class II–IV), or an event within 90 days prior to the screening visit of stroke, transient ischaemic cerebral attack, acute coronary syndrome or hospitalization for worsening heart failure. The full list of exclusion criteria is in Supplementary data, Table S1.

The randomization visit (Visit 2) occurred up to 2 weeks after the screening visit and was defined as baseline. Participant eligibility and clinical status were rechecked at the baseline visit prior to randomization. A complete physical examination was performed at baseline. Participant body weight was also measured. Vital signs (pulse rate and blood pressure) were assessed at the baseline visit. Creatinine-based eGFR, serum potassium and UACR measurements were assessed at a central laboratory. Clinical chemistry and haematology were measured at both the screening and baseline visits. Lipids (low-density lipoprotein, high-density lipoprotein, total cholesterol and triglycerides) were measured at the screening visit only; HbA1c was measured at the baseline visit only (both fasting and not-fasting conditions were permitted for glucose testing).

Participants were randomized 1:1:1 to once-daily 10 or 20 mg finerenone plus 10 mg empagliflozin, once-daily 10 or 20 mg finerenone plus placebo, or once-daily 10 mg empagliflozin plus placebo. Randomization was stratified by eGFR (<60 mL/min/1.73 m^2^, ≥60 mL/min/1.73 m^2^) and UACR (≤850 mg/g, >850 mg/g) values at screening. Investigators and participants are blinded to each participant's assigned study intervention throughout the course of the study. Each study intervention and its matching placebo are identical in appearance (size, shape and colour). The packaging and labelling are designed to maintain the blinding of the investigator's team and the participants. The study data will remain blinded until database lock and authorization of data release according to standard operating procedures.

The starting dose of finerenone was based on the participant's eGFR level at the screening visit: 10 mg once daily if eGFR was <60 mL/min/1.73 m^2^ and 20 mg once daily (target dose) if eGFR was ≥60 mL/min/1.73 m^2^. For participants on the lower dose, this can be uptitrated to 20 mg after 30 days if serum/plasma potassium concentration is ≤4.8 mmol/L and the eGFR decrease is <30% compared with the value measured at the previous visit. Corresponding sham uptitrations will be performed for placebo. Downtitration from a 20-mg to a 10-mg dose is permitted for safety reasons at any time.

The primary efficacy outcome is the relative change in log-transformed UACR from baseline at Day 180. Secondary key outcomes include the proportion of individuals achieving a reduction in UACR >30% from baseline to Day 180, the incidence of hyperkalaemia (moderate hyperkalaemia defined as serum potassium levels ≥5.5 to ≤6.0 mmol/L; severe hyperkalaemia defined as serum potassium levels >6.0 mmol/L) and acute kidney injury [defined as an increase in serum creatinine ≥0.3 mg/dL within 48 h or ≥1.5 times the baseline (known or presumed to have occurred within the prior 7 days), or a urine volume <0.5 mL/kg/hour for 6 h], and the assessment of initial and longer-term changes in eGFR.

Participant analysis sets include the full analysis set (defined as all randomized participants) and the safety analysis set (defined as all randomized participants who received at least one dose of study medication).

The study protocol, protocol amendment and informed consent forms were approved by independent review boards and independent ethics committees according to country-specific requirements. The study was conducted in compliance with the principles of the Declaration of Helsinki and in accordance with the International Conference on Harmonization Guidelines for Good Clinical Practice. All study participants provided written informed consent prior to study enrolment. The study is registered with www.clinicaltrials.gov (NCT05254002; EudraCT 2021-003037-11).

## RESULTS

### Participant disposition

**Figure 2:**
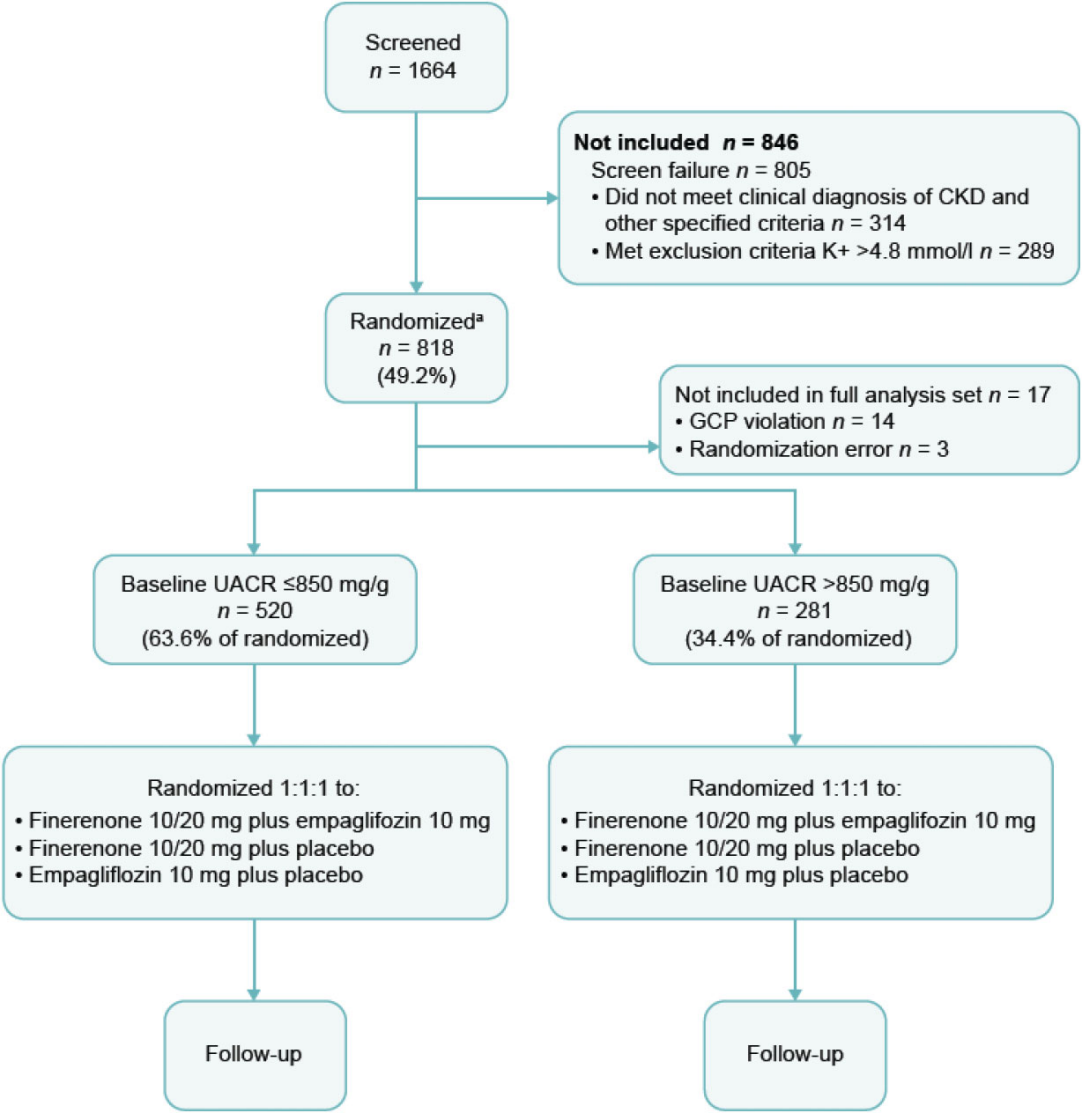
Participant disposition. ^a^Participants were also stratified by eGFR (<60 or ≥60 mL/min/1.73 m^2^). GPC, Good Clinical Practice; K+, potassium.

In total, 1664 participants were screened. A total of 805 participants did not pass screening; of those, 314 (39%) did not meet the inclusion criteria of a clinical diagnosis of CKD as defined by eGFR, UACR and other specified criteria, and 289 (36%) met the exclusion criteria of serum potassium levels >4.8 mmol/L at the screening visit. Of the screened participants, 818 were randomized across 143 sites in 14 countries (Fig. 2). The first participant was randomized in July 2022 and randomization was completed in August 2024. Seventeen participants were excluded from the full analysis set (*N* = 801) due to Good Clinical Practice violations or randomization errors.

The numbers of patients in the lower (≤850 mg/g) and higher (>850 mg/g) UACR categories were 529 and 289, respectively (Fig. 2 and Table 1).

**Table 1:** Baseline demographic and clinical characteristics stratified by UACR.

	UACR group		
Characteristics	≤850 mg/g (*n* = 520)	>850 mg/g (*n* = 281)	Total (*N* = 801)^a^
Age, years, mean ± SD	67.6 ± 9.8	64.3 ± 10.8	66.5 ± 10.3
Age ≥65 years, *n* (%)	347 (66.7)	152 (54.1)	499 (62.3)
Sex, *n* (%)			
Male	390 (75.0)	213 (75.8)	603 (75.3)
Female	130 (25.0)	68 (24.2)	198 (24.7)
Race, *n* (%)^b^			
White	248 (47.7)	104 (37.0)	352 (43.9)
Asian	214 (41.2)	157 (55.9)	371 (46.3)
Asian Indian	72 (13.8)	61 (21.7)	133 (16.6)
Black/African American	50 (9.6)	17 (6.0)	67 (8.4)
Other	2 (0.4)	3 (1.1)	5 (0.6)
Ethnicity, *n* (%)^c^			
Hispanic or Latino	58 (11.2)	25 (8.9)	83 (10.4)
Non-Hispanic or Latino	457 (87.9)	256 (91.1)	713 (89.0)
Region, *n* (%)			
Europe	156 (30.0)	60 (21.4)	216 (27.0)
North America	158 (30.4)	67 (23.8)	225 (28.1)
Asia	206 (39.6)	154 (54.8)	360 (44.9)
Smoking history, *n* (%)			
Current smoker	70 (13.5)	47 (16.7)	117 (14.6)
Former smoker	172 (33.1)	85 (30.2)	257 (32.1)
No history of smoking	278 (53.5)	149 (53.0)	427 (53.3)
eGFR, mL/min/1.73 m^2^, mean ± SD^d^	55.4 ± 17.2	52.0 ± 16.6	54.2 ± 17.1
eGFR category, mL/min/1.73 m^2^, *n* (%)			
>75	76 (14.6)	31 (11.0)	107 (13.4)
60 to 75	118 (22.7)	55 (19.6)	173 (21.6)
<60	326 (62.7)	195 (69.4)	521 (65.0)
UACR, mg/g, geometric mean ± GSD	354 ± 2.2	1397 ± 1.9	574 ± 2.7
UACR, mg/g, median (IQR)	371 (221–592)	1299 (937–2122)	583 (292–1140)
Category of albuminuria, mg/g, *n* (%)			
<30	2 (0.4)^e^	0	2 (0.2)
30 to 300	202 (38.8)	5 (1.8)^c^	207 (25.8)
>300 to 1000	287 (55.2)	78 (27.8)	365 (45.6)
>1000	27 (5.2)	198 (70.5)	225 (28.1)
Missing data	2 (0.2)	0	2 (0.2)
BMI, kg/m^2^, mean ± SD	29.3 ± 6.0	29.3 ± 6.1	29.3 ± 6.1
Body weight, kg, mean ± SD	82.3 ± 20.1	81.4 ± 21.2	82.0 ± 20.5
SBP, mmHg, mean ± SD	135 ± 13	136 ± 14	135 ± 13
DBP, mmHg, mean ± SD	77 ± 9	77 ± 10	77 ± 10
Haemoglobin, g/dL, mean ± SD	13.1 (1.7)	12.6 (1.8)	12.9 (1.8)
HbA1c, %, mean ± SD	7.2 ± 1.2	7.5 ± 1.3	7.3 ± 1.2
Serum potassium, mmol/L, mean ± SD	4.48 ± 0.41	4.48 ± 0.46	4.48 ± 0.42
Medical history, *n* (%)^f^			
Hypertension	458 (88.1)	245 (87.2)	703 (87.8)
Atherosclerotic cardiovascular disease	134 (25.8)	89 (31.7)	223 (27.8)
Coronary artery disease	77 (14.8)	56 (19.9)	133 (16.6)
Myocardial infarction	25 (4.8)	14 (5.0)	39 (4.9)
Stroke	40 (7.7)	21 (7.5)	61 (7.6)
Peripheral arterial disease	32 (6.2)	25 (8.9)	57 (7.1)
Atrial fibrillation	39 (7.5)	12 (4.3)	51 (6.4)
Heart failure	22 (4.2)	8 (2.8)	30 (3.7)
Diabetic retinopathy	70 (13.5)	56 (19.9)	126 (15.7)
Concomitant medications, *n* (%)			
ACEi/ARBs^g^	510 (98.1)	278 (98.9)	788 (98.4)
Calcium channel blockers	304 (58.5)	187 (66.5)	491 (61.3)
Statins	199 (38.3)	114 (40.6)	313 (39.1)
Antiplatelet agents	190 (36.5)	129 (45.9)	319 (39.8)
Beta-blockers	176 (33.8)	103 (36.7)	279 (34.8)
Diuretics	199 (38.3)	92 (32.7)	291 (36.3)
Potassium-lowering agents	4 (0.8)	1 (0.4)	5 (0.6)
Potassium supplements	4 (0.8)	1 (0.4)	5 (0.6)
Antihyperglycaemic agents, *n* (%)			
Insulin	198 (38.1)	115 (40.9)	313 (39.1)
GLP-1 RAs	124 (23.8)	58 (20.6)	182 (22.7)
Oral hypoglycaemic agents			
Metformin	315 (60.6)	166 (59.1)	481 (60.0)
DPP-4 inhibitors	147 (28.3)	102 (36.3)	249 (31.1)
Sulfonylureas	120 (23.1)	69 (24.6)	189 (23.6)

a There were 17 randomized participants excluded from the full analysis set due to Good Clinical Practice violations or randomization errors.

b Race was not reported for six participants.

c Ethnicity was not reported for five participants.

d Calculated by the CKD-EPI creatinine equation [19] with a modification to the equation for Japanese participants [20].

e Stratification was based on UACR at screening. UACR may have decreased for participants between the screening and randomization visits.

f Coded using the MedDRA dictionary.

g According to the protocol, all patients were required to use an ACEi or ARB at the clinically maximum tolerated dose.

BMI, body mass index; DBP, diastolic blood pressure; DPP, dipeptidyl peptidase 4; GSD, geometric standard deviation; IQR, interquartile range; SBP, systolic blood pressure.

### Baseline demographics and clinical characteristics

Baseline demographic and clinical characteristics based on the full analysis set population and displayed by UACR stratification are shown in Table 1. Overall baseline characteristics were similar between the participants in the lower and higher UACR groups.

For the overall study population, the mean [standard deviation (SD)] age was 66.5 (10.3) years and 499 (62.3%) participants were ≥65 years of age. Overall, 603 (75.3%) and 198 (24.7%) of the trial population were male and female, respectively. CONFIDENCE enrolled participants globally, with 360 (44.9%), 225 (28.1%) and 216 (27.0%) enrolled in Asia, North America and Europe, respectively. Overall, 371 (46.3%), 352 (43.9%) and 67 (8.4%) participants were Asian, White and Black/African American, respectively; 83 (10.4%) participants were of Hispanic or Latino ethnicity. At the time of participant consent, 117 (14.6%) and 257 (32.1%) participants were current and former smokers, respectively. A total of 427 (53.3%) participants had never smoked.

Baseline mean ± SD eGFR was 54.2 ± 17.1 mL/min/1.73 m^2^. In addition, the number of participants in eGFR (mL/min/1.73 m^2^) categories <30, ≥30 to <45, ≥45 to <60, ≥60 to 75, and >75 was 27 (3.4%), 264 (33.0%), 230 (28.7%), 173 (21.6%) and 107 (13.4%), respectively. The median (interquartile range) UACR was 583.44 (291.72 to 1140.00) mg/g. There were 2 (0.2%) participants in the albuminuria A1 category and 207 (25.8%) in the albuminuria A2 category. Within the albuminuria A3 category, 365 (45.6%) participants had a UACR >300 to 1000 mg/g, and 225 (28.1%) had a UACR >1000 mg/g. The cohort distribution of KDIGO categories of kidney disease risk is displayed in Supplementary data, Fig. S1.

Mean ± SD body mass index was 29.3 ± 6.1 kg/m^2^. Mean ± SD systolic blood pressure and diastolic blood pressure was 135 ± 13 and 77 ± 10 mmHg, respectively. Mean ± SD HbA1c was 7.3 ± 1.2%. Mean ± SD serum potassium was 4.48 ± 0.42 mmol/L (mEq/L).

### Medication usage

In total, 788 (98.4%) study participants were using an ACEi or ARB. Statins were used in 313 (39.1%) participants, antiplatelet agents in 319 (39.8%) participants and centrally acting antihypertensive drugs in 37 (4.6%) participants. Diuretics were used by 291 (36.3%) and beta-blockers by 268 (33.5%) participants. Diabetes medications included insulin (39.1%) and oral hypoglycaemic agents [sulfonylureas (*n* = 189; 23.6%), dipeptidyl peptidase-4 inhibitors (*n* = 249; 31.1%) and metformin (*n* = 481; 60.0%)]. GLP-1 RAs were used by 182 (22.7%) participants.

### Medical conditions

Atherosclerotic CV disease was present in 223 (27.8%) participants. A history of heart failure prior to screening was present in 30 (3.7%) participants. Diabetic retinopathy was present in 126 (15.7%) participants. Atrial fibrillation was present in 51 (6.4%) participants. Hypertension was present in 703 (87.8%) participants.

### ABPM substudy

The data monitoring committee reviewed (unblinded) ABPM data for participants in Part A. The assessment of safety in the ABPM substudy confirmed there was no elevated hypotension risk in the first 24 h following combined initiation of study medication. Following feedback from the data monitoring committee, the sponsor and the study's steering committee made the decision to move from Part A to Part B of the study, resulting in an extension of enrolment to participants with an eGFR as low as 30 mL/min/1.73 m^2^.

## DISCUSSION

CONFIDENCE is the first randomized clinical trial focused on efficacy in UACR-lowering, and the safety of initiating combination therapy with finerenone and empagliflozin compared with either agent alone, in participants with CKD and T2D. This study will provide insight into the potential additive effect of multiple therapies by examining the change in UACR as a primary outcome measure for clinical benefit.

The CONFIDENCE study is racially and ethnically diverse. There is a high percentage of Asian participants (46.3%), as well as a substantial number of Black/African American participants (8.4%) and participants of Hispanic or Latino ethnicity (10.4%). At baseline, almost all participants (98.4%) were on an ACEi or ARB, and over one-third of participants were on statins or antiplatelet agents. GLP-1 RAs were permitted if the dose was stable for 4 weeks prior to the screening visit, although uptitrations of GLP-1 RAs were not allowed post-randomization (study investigators were encouraged to use insulin or thiazolidinediones for participants requiring improved glycaemic control). Overall, over 20% of participants were on a GLP-1 RA at baseline. Together, these results demonstrate that the CONFIDENCE population is a representative population of people with CKD and T2D and reflects the high quality of care participants received.

Key baseline characteristics of participants in the CONFIDENCE trial include a median UACR of 583 mg/g, a mean eGFR of 54 mL/min/1.73 m^2^ and a mean HbA1c of 7.3%. Kidney disease characteristics in participants with CKD and T2D from other diverse trials are shown in Table 2. While the mean eGFR (mL/min/1.73 m^2^) in CONFIDENCE is similar to that in in CREDENCE [(NCT02065791) 56.2], it is higher than in FIDELIO-DKD (44.3), EMPA-KIDNEY [(NCT03594110) 36.0], DAPA-CKD [(NCT03036150) 43.8], FLOW (47.0) and MOSAIC [(NCT04026165) 35.0], but lower than in FIGARO-DKD (67.8) [21–27]. In addition, the median UACR (mg/g) in CONFIDENCE (583) is similar to that in FLOW (568), higher than in FIGARO-DKD (308) and EMPA-KIDNEY (348), and lower than in FIDELIO-DKD (852), CREDENCE (927), DAPA-CKD (1017) and MOSAIC (986) [21–27].

**Table 2:** Kidney disease characteristics in participants with CKD and T2D by study.

Characteristics	CONFIDENCE (*N* = 801)	FIDELIO-DKD [21] (*N* = 5674)	FIGARO-DKD [25] (*N* = 7352)	CREDENCE [26, 35] (*N* = 4401)	EMPA -KIDNEY [22] (*N* = 3039)^a^	DAPA-CKD [24] (*N* = 2906)^a^	FLOW [27] (*N* = 3533)	MOSAIC [23] (*N* = 310)
eGFR, mL/min/1.73 m^2^, mean ± SD^b^	54.2 ± 17.1	44.3 ± 12.6	67.8 ± 21.7	56.2 ± 18.2	36.0 ± 13.9	43.8 ± 12.6	47.0 ± 15.2	35.0 ± 10.0
eGFR category, mL/min/1.73 m^2^, *n* (%)								
≥75	107 (13.4)							
		656 (11.6)	4539 (61.7)	1809 (41.1)		348 (12.0)	719 (20.4)	
≥60 to <75	173 (21.6)				532 (18.0)			
≥45 to <60	230 (28.7)	1900 (33.5)	1534 (20.9)	1279 (29.1)		918 (31.6)	1055 (29.9)	61 (20.0)
≥30 to <45	264 (33.0)	2981 (52.5)^c^	1251 (17.0)^c^	1313 (29.8)	1359 (45.0)	1239 (42.6)	1358 (38.4)	124 (40.0)
<30	27 (3.4)	135 (2.4)^d^	27 (0.4)^d^	0 (0)	1148 (38.0)	401 (13.8)	400 (11.3)	125 (40.0)
Missing data	0 (0)	2 (<0.1)	1 (<0.1)	0 (0)	0 (0)	0 (0)	1 (<0.1)	0 (0)
UACR, mg/g, median (IQR)	583.44 (291.72–1140.00)	852 (446–1634)	308 (108–740)	927 (463–1833)	348 (68–1293)	1017^e^	568^e^	986 (484–1775)
Category of albuminuria, *n* (%)								
A1, normoalbuminuria (<30 mg/g)	2 (0.2)	23 (0.4)	207 (2.8)	31 (0.7)	649 (21.4)	1 (0.0)	109 (3.1)	-
A2, microalbuminuria (≥30 to ≤300 mg/g)	207 (25.8)	685 (12.1)	3414 (46.4)	496 (11.3)	941 (31.0)	308 (10.6)	1004 (28.4)	-
A3, macroalbuminuria (>300 mg/g)	590 (73.7)	4963 (87.5)	3729 (50.7)	3874^f^ (88.0)	1449 (47.7)	2597 (89.4)	2419 (68.5)	-
Missing data	2 (0.2)	3 (<0.1)	2 (<0.1)	0 (0)	0 (0)	0 (0)	1 (<0.1)	-

a Data are shown for participants with T2D only.

b Calculated by the CKD-EPI equation [19] with a modification to the equation for Japanese participants [20].

c ≥25 to <45 mL/min/1.73 m^2^.

d <25 mL/min/1.73 m^2^.

e IQR not stated.

f UACR ≥300 mg/g.

IQR, interquartile range.

UACR has been identified as a surrogate for CKD progression in clinical trials [28, 29], and is a risk factor for CV events in individuals with or without diabetes [30]. Interventions that reduce elevated levels of UACR lower the risk of those adverse outcomes [31]. Therefore, an argument can be made that interventions for CKD should focus upon strategies that lower UACR to the greatest extent possible. A meta-analysis of studies assessing treatment effects on early changes in albuminuria and clinical outcomes demonstrated that a greater reduction in UACR leads to greater outcomes benefit; for example, a 30% reduction in albuminuria is associated with a 27% reduction in kidney outcomes [29]. A mediation analysis using pooled data from FIDELIO-DKD and FIGARO-DKD demonstrated that finerenone-induced reductions in UACR (analysed as a continuous variable) mediated 84% of the treatment effect on kidney outcomes [32]. In addition, change in UACR with finerenone mediated 37% of the treatment effect for CV outcomes [32]. Moreover, mediation analyses of SGLT2is (evaluated in univariate analyses) demonstrated that UACR mediated the treatment effect of empagliflozin on kidney outcomes by 31% in the EMPA-REG OUTCOME trial [33], and canagliflozin on kidney outcomes by 24% in the CANVAS Program [34]. Of note, in the CANVAS Program, the mediating effects of UACR were greatly associated with the baseline UACR level; UACR mediated 42% of the canagliflozin effect on the kidney endpoint in participants with a baseline UACR ≥30 mg/g, and 7% in those with a baseline UACR <30 mg/g [34]. Together, these findings highlight the importance of targeting UACR reduction as a therapeutic target in the treatment of CKD in people with T2D.

In summary, the high rates of morbidity and mortality associated with CKD in T2D highlight the unmet need for additional effective treatments for slowing CKD progression. People with both CKD and T2D are at high risk of adverse clinical outcomes [6] because of the additive, detrimental effects of these conditions. CONFIDENCE is the first trial to examine combination therapy comprising finerenone and an SGLT2i in people with CKD and T2D, and will provide the evidence to determine the potential role of simultaneous initiation of finerenone and SGLT2is to better inform the care of people in this population.

## Supplementary Material

gfaf022_Supplemental_File

## Data Availability

Availability of the data underlying this publication will be determined according to Bayer's commitment to the EFPIA/PhRMA ‘Principles for responsible clinical trial data sharing’. This pertains to scope, time point and process of data access. As such, Bayer commits to sharing, upon request from qualified scientific and medical researchers, patient-level clinical trial data, study-level clinical trial data and protocols from clinical trials in patients for medicines and indications approved in the USA and the EU as necessary for conducting legitimate research. This applies to data on new medicines and indications that have been approved by the EU and US regulatory agencies on or after 1 January 2014. Interested researchers can use www.vivli.org to request access to anonymized patient-level data and supporting documents from clinical studies to conduct further research that can help advance medical science or improve patient care. Information on the Bayer criteria for listing studies and other relevant information is provided in the member section of the portal. Data access will be granted to anonymized patient level data, protocols and clinical study reports after approval by an independent scientific review panel. Bayer is not involved in the decisions made by the independent review panel. Bayer will take all necessary measures to ensure that patient privacy is safeguarded.
