# Supplementary material for: COmbinatioN effect of FInerenone anD EmpaglifloziN in participants with chronic kidney disease and type 2 diabetes using a UACR Endpoint (CONFIDENCE) trial: baseline clinical characteristics
Source: Nephrol Dial Transplant. 2025 Feb 7;40(8):1559–69. doi: 10.1093/ndt/gfaf022 (PMC12315800; doi:10.1093/ndt/gfaf022)
Supplement: gfaf022_Supplemental_File [file gfaf022_supplemental_file.docx]

Supplementary appendix

Supplement to: Agarwal R, Green JB, Heerspink HJL, *et al*. COmbinatioN effect of FInerenone anD EmpaglifloziN in participants with chronic kidney disease and type 2 diabetes using a UACR Endpoint (CONFIDENCE) trial: Baseline clinical characteristics. This appendix has been provided by the authors to give readers additional information about the work.

Table of Contents

[Supplementary appendix 1](#_Toc187333640)

[Investigators by country 3](#_Toc187333641)

[Committees and study personnel 11](#_Toc187333642)

[Steering committee 11](#_Toc187333643)

[Data monitoring committee 11](#_Toc187333644)

[National lead investigators 11](#_Toc187333645)

[Table S1 Study exclusion criteria 13](#_Toc187333646)

[Figure S1: Distribution of KDIGO risk categories stratified by UACR <850 mg/g (A) and ≥850 mg/g (B). 14](#_Toc187333647)

[SUPPLEMENTARY REFERENCES 15](#_Toc187333648)

# Investigators by country

**Belgium**

An Nollet, Jan Yperman Ziekenhuis, Ieper

Bruno Van Vlem, Onze-Lieve-Vrouwziekenhuis VZW, Aalst

Francis Duyck, AZ Delta - Campus Rumbeke, Roeselare

Marijn Speeckaert, Universitair Ziekenhuis Gent, Gent

Peter Doubel, AZ Groeninge, Kortrijk

Pieter Gillard, Universitair Ziekenhuis Leuven, Leuven

**Canada**

Elie Sahyouni, LMC Diabetes & Endocrinology Ville St Laurent, Saint-Laurent

Giuseppe Mazza, GCP Trials, Montreal

Hitesh Mehta, Regional Kidney Wellness Centre, Brampton

Richard Tytus, Hamilton Medical Research Gr, Hamilton

Sameh Fikry, Dr. Sameh Fikry Medicine Professional Corporation, Waterloo

Sean Peterson, Bluewater Clinical Research Group, Sarnia

Shivinder Jolly, Clinical Research Solutions, Waterloo

William Beaubien-Souligny, Centre hospitalier de l'Université de Montréal, Montreal

**Denmark**

Claus Juhl, Sydvestjysk sygehus, Esbjerg

Jesper Nørgaard Bech, Regionshospitalet Gødstrup, Holstebro

Peter Rossing, Steno Diabetes Center, Herlev

Thure Krarup, Bispebjerg Hospital, København Nv

**France**

Bruno Guerci, Centre Hospitalier Régional Universitaire de Nancy - Hôpitaux de Brabois, Vandoeuvre-lès-Nancy

Bruno Verges, Centre Hospitalier Universitaire Dijon, Dijon

Jean-Pierre Fauvel, Centre Hospitalier Universitaire de Lyon, Lyon

Olivier Dupuy, Hôpital Paris Saint Joseph, Paris

Olivier Moranne, Hôpital Universitaire Carémeau, Nîmes, and Hôpital Robert Debré AP-HP, Paris

Pierre-Louis Carron, Centre Hospitalier Universitaire Grenoble Alpes-Hôpital Nord Michallon, La Tronche

**Germany**

Bernhard Winkelmann, ClinPhenomics GmbH & Co, Frankfurt

Christof Kloos, Universitätsklinikum Jena Klinik, Jena

Christoph Axthelm, Cardiologicum Dresden and Pirna, Dresden

Klaus Busch, Gemeinschaftspraxis Diabetesz., Dortmund

Lutz Stemler, Die Praxis am Ludwigsplatz, Ludwigshafen

Markus van der Giet, Charité – Universitätsmedizin Berlin, Berlin

Thorsten Koch, Diabetes Zentrum Wandsbek, Hamburg

**India**

Architkumar Patel, Vedanta Kidney Care, Vadodara

Balasubramaniyan Thopplan, Government Kilpauk Medical College Hospital, Chennai

Chandrashekar Matad, Bangalore Medical College & Research Institute, Bangalore

Dinesh Khullar, Max Super Speciality Hospital Saket, Delhi

Ganapathi Bantwal, St. John’s Medical College and Hospital, Bengaluru

Hansraj Alva, Vinaya Hospital, Mangalore

Jayakumar EK, Government Medical College, Kozhikode

Jugal Bihari Gupta, Eternal Heart Care Centre and Research Institute, Jaipur

Keshavamurthy CB, Manipal Hospital Mysore, Mysore

Manisha Sahay, Osmania General Hospital, Hyderabad

Pal Atanu, Institute of Post-Graduate Medical Education and Research and Seth Sukhlal Karnani Memorial Hospital , Kolkata

Prabha Dadala Ratna, King George Hospital, Visakhapatnam

Sameer Chaubey, Asian Kidney Hospital & Medical Centre, Nagpur

Sanjay Chunilal Agarwal, Grant Medical Foundation, Pune

Saurabh Agarwal, Ganesh Shankar Vidyarthi Memorial Medical College - Kanpur, Kanpur

Sharma Balram, Sawai Man Singh Medical College Hospital, Jaipur

Siddharth Mavani, Mavani Research Center, Ahmedabad

Sree Bhushan Raju, Nizam's Institute of Medical Sciences, Hyderabad

Sreedhar Reddy, Krishna Institute of Medical Sciences, Secunderabad

Subhash Wangnoo, Indraprastha Apollo Hospital, New Delhi

Tushar Bandgar, King Edward Memorial Hospital, Mumbai

Vernekar Ritesh, KLES Dr Prabhakar Kore Hospital, Belagavi

**Israel**

Faiad Adawi, Ziv Medical Center, Safed

Genya Aharon-Hananel, Hadassah Medical Center, Jerusalem

Idit Liberty, Soroka Medical Center, Be’er-Sheva

Julio Wainstein, Edith Wolfson Medical Center, Holon

Mazen Elias, Emek Medical Center, Afula

Nimer Assy, Galilee Medical Center, Nahariya

Nomy Levin-Iaina, Barzilai Medical Center, Ashkelon

**Italy**

Agostino Consoli, Centro di Ricerca Clinica, Chieti

Anna Maria Grazia Veronelli, ASST Santi Paolo e Carlo, Milan

Emanuele Bosi, IRCCS Ospedale San Raffaele, Milan

Enrico Fiaccadori, Azienda Ospedaliero-Universitaria di Parma, Parma

Giancarlo Tonolo, Azienda Sanitaria Locale Olbia – Ospedale S. Giovanni di Dio, Olbia

Giuseppe Penno, Azienda Ospedaliero Universitaria Pisana - Stabilimento di Santa Chiara, Pisa

Paola Ponzani, Azienda Sanitaria Locale, Chiavari, Genoa

Roberta Poli, San Luigi Gonzaga University Hospital, Orbassano

Roberto Cimino, Ospedale Garbagnate Milanese, Garbagnate Milanese

Roberto Trevisan, ASST Papa Giovanni XXIII, Bergamo

Salvatore De Cosmo, Fondazione Casa Sollievo della Sofferenza, San Giovanni Rotondo

Veronica Resi, Policlinico Maggiore di Milano, Milan

**Japan**

Daishiro Yamada, Jiyugaoka Yamada International Medicine Clinic, Obihiro-Shi

Fumi Umeoka, Saiseikai Matsuyama Hospital, Matsuyama-Shi

Hideo Kanehara, Fukui-ken Saiseikai Hospital, Fukui-Shi

Hidetoshi Kanai, Kokura Memorial Hospital, Kitakyushu-Shi

Kunihisa Kobayashi, Fukuoka University, Chikushino

Masahiko Ochi, Public Central Hospital of Matto Ishikawa, Ishikawa

Masao Ishii, Fukuoka Wajiro Hospital, Fukuoka-Shi

Takeshi Osonoi, Nakakinen Clinic Med Corporation, Naka-Shi

Terumasa Hayashi, Osaka General Medical Center, Osaka-Shi

Yoshihide Hirohata, Hirohata Naika Clinic, Kitakyushu-Shi

Yoshimitsu Yamasaki, Kyosokai AMC Nishi-Umeda Clinic, Osaka-Shi

**Netherlands**

Jeroen van der Net, Albert Schweitzer Ziekenhuis, Dordrecht

Mirjam Lips, Onze Lieve Vrouwe Gasthuis, Amsterdam

Paul Rootjes, Gelre Ziekenhuizen, Apeldoorn

Peter Luik, Meander Medisch Centrum, Amersfoort

**Republic of Korea**

Byung Wan Lee, Yonsei University College of Medicine, Seoul

Chang Beom Lee, Hanyang University Guri Hospital, Guri-si

Choon-Hee Chung, Yonsei University, Wonju-si, Gangwon-do

EunYoung Lee, Soon Chun Hyang University Cheonan, Incheon

Jae-Myung Yu, Hallym University Kangnam Sacred Heart Hospital, Seoul

Seok Joon Shin, Incheon St. Mary's Hospital, Incheon

Soo Lim, Seoul National Bundang Hospital, Seongnam-si

Sung-Gyun Kim, Hallym University Sacred Heart Hospital, Anyang-si

Woo-Je Lee, Asan Medical Center, Seoul

You-Cheol Hwang, Kyung Hee University Hospital Gangdong, Seoul

Young Min Cho, Seoul National University Hospital, Seoul

Young Sun Kang, Korea University Ansan Hospital, Ansan City

**Spain**

Alberto Ortiz Arduan, Hospital Universitario Fundación Jiménez Díaz, Madrid

Alfonso Soto, Complexo Hospital University A Coruña, A Coruña

Cristina Castro, Hospital Universitario Dr. Peset, Valencia

Cristobal Morales, Hospital Vithas Sevilla, Castilleja De La Cuesta

Fernando Cereto Castro, Hospital Quirónsalud Barcelona, Barcelona

Francisco Martinez Deben, Complexo Hospitalario Universitario de Ferrol, Ferrol

Francisco Tinahones Madueno, Hospital Universitario Virgen de la Victoria, Malaga

Hanane Bourarich, Hospital Universitario Príncipe de Asturias, Alcala de Henares

Jose Luis Górriz Teruel, Hospital Clínico Universitario de Valencia, Valencia

Juan Diego Mediavilla, Hospital Universitario Virgen de las Nieves, Granada

Maria Jose Soler Romeo, Hospital Universitario Vall d'Hebron, Barcelona

Maria Marques Vidas, Hospital Universitario Puerta de Hierro Majadahonda, Majadahonda

**Taiwan**

Chien-Te Lee, Chang Gung Memorial Hospital, Kaohsiung City

Chiz-Tzung Chang, China Medical University Hospital, Taichung

Der-Cherng Tarng, Taipei Veterans General Hospital, Taipei

Ju-Ying Jiang, Far Eastern Memorial Hospital, New Taipei City

Mai-Szu Wu, Taipei Medical University Shuang Ho Hospital, New Taipei City

Ming Ju Wu, Taichung Veterans General Hospital, Taichung

Shih-Te Tu, Changhua Christian Hospital, Changhua

**United States of America**

Ahmed Awad, Clinical Research Consultants, LCC, Kansas City

Ali Iranmanesh, Salem VA Medical Center, Salem

Amy Mottl, University of North Carolina Kidney Center, Chapel Hill

Ankur Doshi, Victorium Clinical Research, Houston

Ashar Luqman, Renal Associates PC., Dakota Dunes

Bruce Baker, North Texas Kidney Disease Ass, Lewisville

Carol Wysham, MultiCare Rockwood Main Clinic, Spokane

Carolina Solis-Herrera, First Outpatient Research Unit, San Antonio

Csaba Kovesdy, University of Tennessee Health Science Center, Memphis

Dana Mitchell, Global Kidney Center, Houston

David Gaskin, Meridian Clinical Research, Savannah

David LaMond, Blue Sky MD, Hendersonville

German Hernandez, DaVita Clinical Research, El Paso

Gloria Ortiz, Biopharma Informatic LLC - McAllen, McAllen

Guillermo Umpierrez, Emory University School of Medicine, Atlanta

Harold Miller, Crescent City Clinical Research, Metairie

Harvey Serota, St. Louis Heart and Vascular, St. Louis

Iqbal Khalid, Southeast Kidney Associates, East Point

Jared Probst, Olympus Family Medicine, Holladay

Jay Sandberg, Oakland Medical Research, Troy

Jay Shubrook, Touro University California, Vallejo

Jose Mandry, West Orange Endocrinology, Ocoee

Joseph Ravid, Innovative Research Institute, Port Charlotte

Julie Silverstein, Washington University in St. Louis School of Medicine, St. Louis

Keung Lee, Carolina Clinical Research & Consulting at Triad Internal Medicine, Asheboro

Leslie Spry, Somnos Sleep Disorders Center, Lincoln

Mariana Garcia-Touza, Kansas City VA Medical Center, Kansas City

Minesh Rajpal, Southwest Kidney Institute, Surprise

Mohamed El-Shahawy, Academic Medical Research Institute, Los Angeles

Nauman Shahid, Eastern Nephrology Associates - Greenville, Greenville

Osvaldo Brusco, Office of Osvaldo A. Brusco, Corpus Christi

Pablo Pergola, Clinical Advancement Center, San Antonio

Pedro Velasquez Mieyer, Dar Salud Care, PLLC - LifeDoc, Memphis

Piotr Lazowski, South Shore Nephrology, Plymouth

Raj Singh, HEALOR Primary Care, Las Vegas

Rekha John, Eastern Nephrology Associates - Kinston Office, Kinston

Richard Powell, Velocity Clinical Research, Cincinnati

Scott Hines, Crystal Run Healthcare, Middletown

Steve Fordan, Thyroid, Endocrinology, and Diabetes, Dallas

Syed Pervaiz, Santa Rosa Medical Center of Nevada, Las Vegas

Tuan-Huy Tran, Crescent City Clinical Research, Metairie

Wajdi Al-Shweiat, Lake Michigan Nephrology, PLC, Saint Joseph

Wayne Kotzker, Florida Kidney Physicians, Boca Raton

William Kaye, Metabolic Research Institute Inc., West Palm Beach

William Yang, Northridge Hospital Medical Center, Granada Hills

# Committees and study personnel

## Steering committee

Rajiv Agarwal, MD, MS, MBBS, FASN (Chair), Indiana University School of Medicine; Janet B. McGill, MD, Washington University in St. Louis; Amy K. Mottl, MD, UNC School of Medicine; Johannes F.E. Mann, MD, KfH Kidney Centre Munich and Friedrich Alexander University; Peter Rossing, MD, Steno Diabetes Center Copenhagen and University of Copenhagen; Masaomi Nangaku, MD, PhD, The University of Tokyo Graduate School of Medicine; Jennifer B. Green, MD, Duke University School of Medicine; George Bakris (former member of the Steering Committee), MD, University of Chicago Medicine; Hiddo J.L. Heerspink, PhD, University Medical Centre Groningen; Julio Rosenstock, MD, Velocity Clinical Research at Medical City; Muthiah Vaduganathan, MD, MPH, Brigham and Women's Hospital and Harvard Medical School

## Data monitoring committee

William B. White, MD (Chair), University of Connecticut Health Center; Tim Friede, PhD, University Medical Center Göttingen; Patrick Rossignol, MD, Princess Grace Hospital Monaco and Monaco Private Hemodialysis Center

## National lead investigators

**Belgium**, Pieter Gillard, University Hospitals Leuven, Leuven

**Denmark**, Jesper Nørgaard Bech, Gødstrup Hospital, Holstebro

**France**, Olivier Moranne, Hôpital Universitaire Carémeau, Nîmes, and Hospital Robert Debré Ap-Hp, Paris

**Germany**, Christoph Axthelm, Cardiologicum Dresden and Pirna, Dresden

**India**, Subhash Wangnoo, Indraprastha Apollo Hospital, New Delhi

**India**, Sree Bhushan Raju, Nizam's Institute of Medical Sciences, Hyderabad

**Italy**, Emanuele Bosi, IRCCS Ospedale San Raffaele, Milano

**Spain**, Jose Luis Górriz Teruel, Hospital Clinico Universitario de Valencia, Valencia

**Taiwan**, Mai-Szu Wu, Taipei Medical University-Shuang Ho Hospital, Taipei

Table S1: Study exclusion criteria

| **Exclusion criteria** |
| --- |
| - Type 1 diabetes - Known allergies to finerenone or any SGLT2i - Hepatic insufficiency classified as Child–Pugh class C - BP at Day 1 higher than 160 mmHg SBP or 100 mmHg DBP or SBP lower than 90 mmHg - Bilateral clinically relevant kidney artery stenosis (>75%) or other non-diabetic kidney disease - Kidney allograft in place or a scheduled kidney transplant - Experienced AKI within 6 months prior to screening - Experienced ketoacidosis in the past 5 years - Primary adrenal insufficiency (Addison’s disease) - Stroke, transient ischaemic cerebral attack, acute coronary syndrome (MI, CABG, primary PCI) or hospitalization for worsening HF within 90 days prior to the screening visit - Clinical diagnosis of chronic HFrEF and persistent symptoms (New York Heart Association class II–IV) at screening visit^a^ - Major surgery (major according to the investigator’s assessment) performed within 90 days prior to the screening visit, or scheduled major elective surgery (e.g., hip replacement) within 90 days after screening visit - GI surgery or GI disorder that could interfere with absorption of trial medication in the investigator’s opinion - Any other history, condition, therapy or uncontrolled intercurrent illness (including AKI) which could in the opinion of the investigator affect participant safety compliance with study requirements - Currently treated with an SGLT2i or SGLT1/2i or have received an SGLT2i or SGLT1/2i that cannot be discontinued at least 8 weeks prior to the screening visit and during study intervention treatment - Treated with strong CYP3A4 inhibitors or inducers that cannot be discontinued 7 days before the Day 1 visit - Treated with another MRA (e.g., eplerenone, esaxerenone, spironolactone, canrenone), a renin inhibitor, potassium supplements, a potassium-sparing diuretic (e.g., amiloride, triamterene), a potassium binder agent, or an angiotensin receptor–neprilysin inhibitor within 8 weeks prior to the screening visit and during study intervention treatment^b^ - Currently treated or were treated with finerenone within 8 weeks prior to the screening visit - Participation in another clinical trial with an investigational product within 1 month prior to the screening visit^c^ - Serum potassium above 4.8 mmol/L at screening (central laboratory value). Note: One reassessment of serum potassium is allowed at the screening visit - ALT or AST >3× ULN at screening visit - Breastfeeding female participant - Participant known for lack of compliance with clinic visits or prescribed medication |

^a^ Class 1a recommendation for MRAs

^b^ K+ supplements and potassium binder agents will be allowed during the study for safety reasons

^c^ Participants who received a COVID-19 vaccine whilst still under Emergency Use Utilization will be eligible, provided vaccination occurred at least 1 month prior to screening visit.

AKI, acute kidney injury; ALT, alanine aminotransferase; AST, aspartate aminotransferase; BP, blood pressure; CABG, coronary artery bypass graft; COVID-19, coronavirus disease 2019; CYP3A4, cytochrome P450 isoenzyme 3A4; DBP, diastolic blood pressure; GI, gastrointestinal; HbA1c, glycated haemoglobin; HF, heart failure; HFrEF, heart failure with reduced ejection fraction; K+, potassium; MI, myocardial infarction; MRA, mineralocorticoid receptor antagonist; PCI, percutaneous coronary intervention; SBP, systolic blood pressure, SGLT1/2i, combined sodium–glucose cotransporter 1 and 2 inhibitor; SGLT2i, sodium–glucose cotransporter 2 inhibitor; ULN, upper limit of normal.

Figure S1: Distribution of KDIGO risk categories stratified by UACR <850 mg/g (A) and ≥850 mg/g (B).


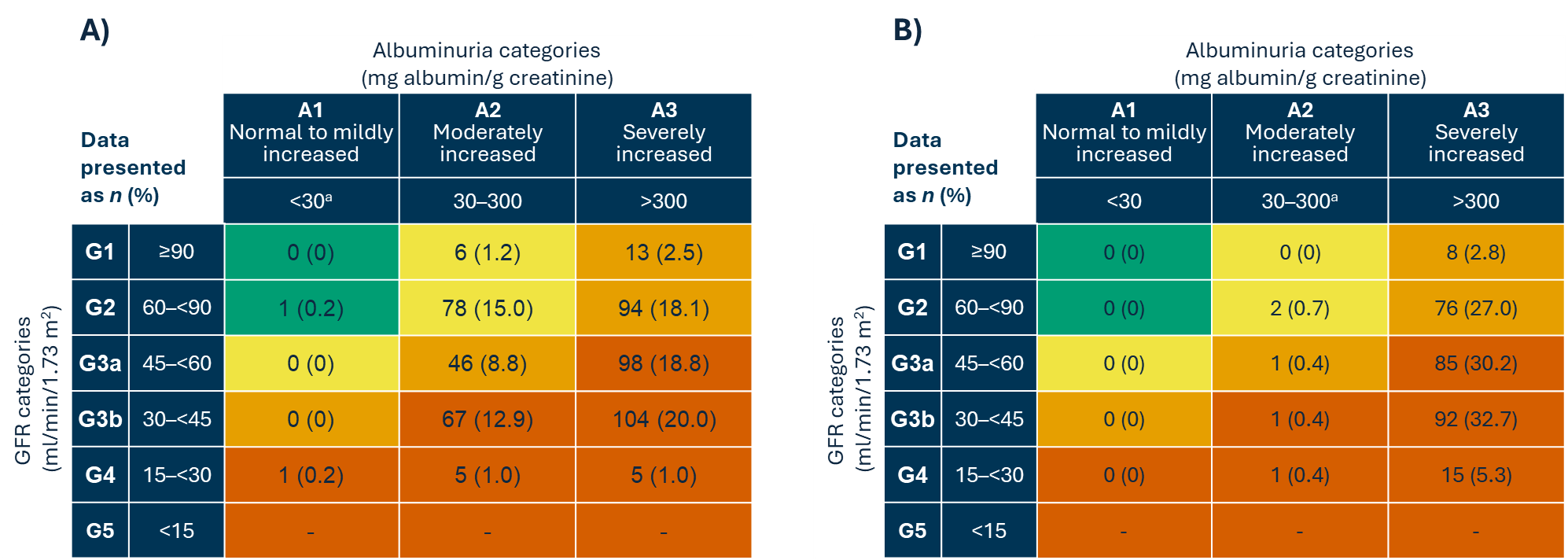


^a^ Stratification was based on UACR at screening. UACR may have decreased for participants between the screening and randomization visits.

A, albuminuria; CKD, chronic kidney disease; G, grade; GFR, glomerular filtration rate; KDIGO, Kidney Disease: Improving Global Outcomes; UACR, urine albumin-to-creatinine ratio.

Adapted from the KDIGO guideline [1] under the terms of the [CC BY-NC-ND 4.0](https://creativecommons.org/licenses/by-nc-nd/4.0/) license.

# SUPPLEMENTARY REFERENCES

1. Kidney Disease: Improving Global Outcomes (KDIGO) CKD Work Group. KDIGO 2024 clinical practice guideline for the evaluation and management of chronic kidney disease. *Kidney Int* 2024;**105**:S117–S314
